# Role of Lipid Rafts in Hematopoietic Stem Cells Homing, Mobilization, Hibernation, and Differentiation

**DOI:** 10.3390/cells8060630

**Published:** 2019-06-22

**Authors:** Munther Alomari, Dana Almohazey, Sarah Ameen Almofty, Firdos Alam Khan, Mohammad Al hamad, Deena Ababneh

**Affiliations:** 1 Department of Stem Cell Biology, Institute for Research and Medical Consultations, Imam Abdulrahman Bin Faisal University, Post Box No. 1982, Dammam 31441, Saudi Arabia; daaalmohazey@iau.edu.sa (D.A.); saalmofty@iau.edu.sa (S.A.A.); fakhan@iau.edu.sa (F.A.K.); 2 Department of Pathology, College of Medicine, Imam Abdulrahman Bin Faisal University, Post Box No. 1982, Dammam 31441, Saudi Arabia; mhamad@iau.edu.sa; 3 Department of Basic Sciences and Humanities, College of Engineering, Imam Abdulrahman Bin Faisal University, Post Box No. 1982, Dammam 31441, Saudi Arabia; dababneh@iau.edu.sa

**Keywords:** LRs, HSCs, differentiation, homing, hibernation, mobilization, SCF, TGF-β, PLC-β2, G-CSF

## Abstract

Hematopoietic stem cells (HSCs) are multipotent, self-renewing cells that can differentiate into myeloid or lymphoid cells. The mobilization and differentiation processes are affected by the external environment, such as extracellular matrix and soluble molecules in the niche, where the lipid rafts (LRs) of the HSCs act as the receptors and control platforms for these effectors. LRs are membrane microdomains that are enriched in cholesterol, sphingolipid, and proteins. They are involved in diverse cellular processes including morphogenesis, cytokinesis, signaling, endocytic events, and response to the environment. They are also involved in different types of diseases, such as cancer, Alzheimer’s, and prion disease. LR clustering and disruption contribute directly to the differentiation, homing, hibernation, or mobilization of HSCs. Thus, characterization of LR integrity may provide a promising approach to controlling the fate of stem cells for clinical applications. In this review, we show the critical role of LR modification (clustering, disruption, protein incorporation, and signal responding) in deciding the fate of HSCs, under the effect of soluble cytokines such as stem cell factor (SCF), transforming growth factor- β (TGF-β), hematopoietic-specific phospholipase Cβ2 (PLC-β2), and granulocyte colony-stimulating factor (G-CSF).

## 1. Introduction

Lipid rafts (LRs) were characterized as functional domains at the plasma membrane more than 30 years ago [1]. Although the existence and biological function of LRs remained controversial for many years, the analysis of their structure and function is currently at the leading edge of biomembrane and cell biology investigations [2,3]. LRs are specialized microdomains (10–200 nm) located in the plasma membrane [3], highly heterogeneous and enriched with sphingolipid (sphingomyelin), sterol, cholesterol, and protein [4,5,6,7,8]. The structure and saturated hydrocarbon chain of sphingolipids allow preferential interaction with cholesterol. In addition, saturated fatty acids and cholesterol can pack tightly, forming a thick and rigid raft area at the cell surface [9,10]. In contrast, the plasma membrane is mainly composed of unsaturated fatty acids and lipids, making it loosely packed and more fluid [11]. The modification of some types of proteins, such as src family kinases and proteins with GPI (glycosylphosphatidylinositol) anchors by fatty acid addition, allows them to associate with LRs [12,13]. LRs are dynamic structures which are able to move through the plasma membrane, collapsing into tiny domains or clustering to form a large platform. They play an interesting role in numerous cellular activities, such as endocytic and secretory pathways [14]. In addition, LRs facilitate cell signaling in B- [15,16] and T-cells [6,17,18]. Several cell surface antigens are either located in, or move into, LRs to be functional [19,20]. For example, the colocalization of the B-cell receptor and CD5 in LRs is necessary to induce the early signaling pathway that leads to apoptosis [21].

Proteins are recruited into LRs as a result of several stimuli (e.g., therapeutic antibodies [22], radiation [23], and hormones [24]). This incorporation is facilitated by a GPI anchor or acylation of these proteins [25]. LRs act as cellular portals that link the exogenous environment to the endocytic pathways and enable the internalization of toxins [26] and viral particles [27]. The allocation of LRs at the cell membrane relies upon cell type. For example, in B- lymphocytes, LRs are accumulated in microvilli-rich regions [28].

LRs are involved in cell membrane trafficking including membrane budding, polarization, invadopodia, and release of exosomes [29,30]. In addition, they have been involved in various diseases, for example, cancer, prion diseases, and Alzheimer’s [31]. The dysregulation of LR proteins results in subsequent effects on signaling and tumor progression. For example, depletion of cholesterol disturbs LR association with prion protein, PrP^C^, and stops PrP^Sc^ conversion. In addition, reorganization of LR cholesterol decreases Aβ formation in Alzheimer’s [32]. This membrane domain also is an important binding site for therapeutic antibodies, for example, anti-CD20 (Rituximab) used to treat CLL patients [33]. In addition, several anticancer drugs have been shown to suppress growth and induce apoptosis of tumor cells through LR remodeling, such as Edelfosine [34], avicin D [35], resveratrol [36], and liver X receptors [37]. Raft microdomains provide a signaling pathway platform capable of various cellular pro- and antiapoptotic pathways that may be initiated upon LR distribution [38,39]. Indeed, many receptor tyrosine kinases are localized in LRs [6,40,41,42,43], highlighting the importance of this membrane microdomain in cell signaling. Furthermore, the modulation of cholesterol levels in acute myeloid leukemia (AML) cells kills and sensitizes them to therapeutic drugs [38,39].

**Figure 1:**
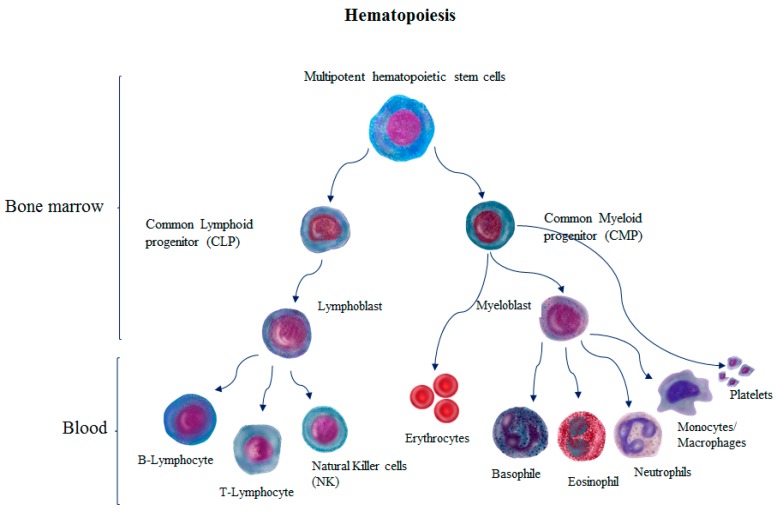
Hematopoiesis process. This process leads to the formation of highly specialized circulating blood cells from HSCs in the bone marrow (BM). Multipotent hematopoietic stem cells in BM differentiate into myeloid or lymphoid progenitor cells. Myeloid cells differentiate into red blood cells, platelets, and myeloblasts, which differentiate into basophils, neutrophils, eosinophils, and macrophages, while lymphoid cells differentiate into B and T-lymphocytes and natural killer cells (NK).

LRs play an important role in hematopoietic stem cells (HSCs) differentiation, homing, and mobilization. HSCs are multipotent, rare, and self-renewing cells that can be found in the bone marrow, umbilical cord blood, and peripheral blood. Most are quiescent at any given time (G0). HSCs were first identified in the dorsal aorta and urogenital ridges (UGR) and characterized with CD45^+^, CD34^+^, VE-cadherin^+^, C-KIT^+^, THY-1^+^, RUNX1^+^, Endoglin^+^, CD45RA^−^, and CD38^−/lo^ [44]. During embryogenesis, HSCs are located in the yolk sac, and then they migrate to the bone marrow, liver, and spleen [45]. HSCs differentiate into all functional types of blood cells of lymphoid and myeloid lineages [46] (Figure 1) and maintain multilineage hematopoiesis throughout the entire lifespan [47,48]. Hematopoiesis or clinical transplantation requires successful mobilization, homing, and differentiation processes of HSCs. Many factors play a role in these processes; here, we focus on the role of LRs.

## 2. HSC Mobilization and Homing

HSCs are located in a specialized place in the niche of bone marrow, with stromal cells, extracellular matrix (ECM), and soluble factors such as cytokines, that control the fate of stem cells [49]. ECM molecules, such as fibronectin, laminin, and collagen, can influence adhesion, maintenance, proliferation, migration, and differentiation of stem cells [50,51]. Interactions between fibronectin and HSCs are mediated through integrins at the cell surface for cell adhesion function [52]. In addition, integrins serve as major receptors for ECM proteins and are capable of mediating bidirectional signal transduction across the plasma membrane [53]. For example, in adherent cells, integrins target Rac protein to the plasma membrane and couple it to the downstream effector, p21-activated kinase, to activate the downstream signaling pathway [54,55]. For integrins to perform the interaction with fibronectin, Rac must be targeted to, and activated by, LRs [56,57]. The disruption of LRs results in prevention of cell adhesion and targeting of Rac to the plasma membrane [58].

**Figure 2:**
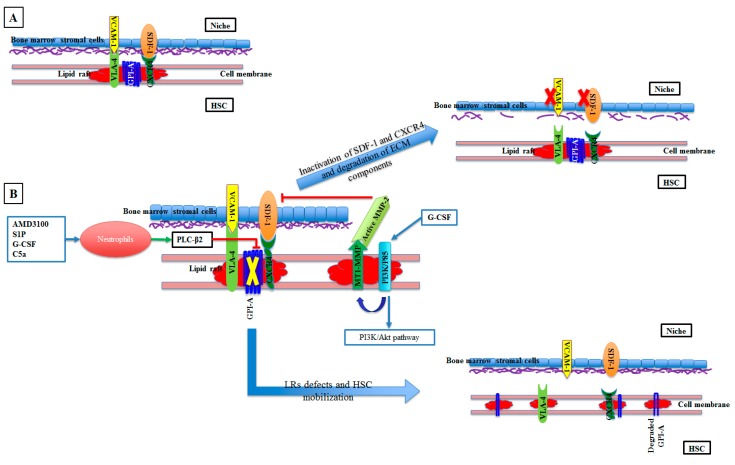
Role of LRs in HSC mobilization. **A**, Quiescent status of HSC in the niche: The LR incorporated proteins VAL-4 and CXCR4 of HSCs bind to VCAM-1 and SDF-1 proteins of the niche, resulting in HSC stabilization. **B**, Mobilization status of HSCs: premobilization factors found in the niche, such as AMD3100, S1P, G-CSF, and C5a, enhance the expression of PLC-β2 of neutrophils. PLC-β2 cleaves GPI-A in the LRs, resulting in disruption of LRs and associated proteins VLA-4 and CXCR4, leading to the detachment of SDF-1 and VCAM-1 ligands and HSC mobilization. In addition, G-CSF activates PI3K/P85 located in the LRs of HSCs, leading to activation of the PI3K/Akt pathway and incorporation of MT1-MMP into the LR, activating MMP-2 at the cell surface. MMP-2 then modulates the matrix, inactivating SDF-1 and CXCR4 and enhancing pericellular degradation of the ECM components. These changes result in the release of HSCs from the bone marrow niches.

LR structure on the surface of the cell membrane helps the association of signaling molecules such as Rac-1, Lyn, and RhoH, with the LR-associated surface receptors CXCR4, α1β4 integrin (VLA-4), and CD 117. This results in regulation of the migration, mobilization, and homing of HSCs. VLA-4 and CXCR4 are LR-associated proteins that bind to ligands VCAM-1 and SDF-1, respectively. These ligands are expressed within the niche environment of stem cells and are consequently involved in anchoring the HSCs to the niches [59,60,61,62]. At the mobilization stage, PLC-β2 is expressed by neutrophils in response to premobilization factors such as AMD3100, sphingosine 1-phosphate (S1P), G-CSF, and C5a. PLC-β2 cleaves glycolipid glycosylphosphatidylinositol anchor (GPI-A), which is necessary for LR integrity, partitioning, signal transduction, and cellular communication. Cleavage of GPI-A results in the disruption of LR and associated proteins VLA-4 and CXCR4, leading to the detachment of SDF-1 and VCAM-1 ligands and hence to HSC mobilization. Also, PLC-β2 removes VCAM-1, CD59, and CD55 from the cell membrane, resulting in activation of the mobilization-promoting complement cascade [59,63], as shown in Figure 2. On the other hand, exposure of HSCs to heat treatment (39.5 °C) leads to the incorporation of CXCR4 into functional LRs with Rac1, which subsequently enhances HSCs homing and engraftment [64].

Successful HSC transplantation requires modulation processes such as mobilization and homing, which are controlled by membrane type 1 metalloprotease (MT1-MMP). The presentation of MT1-MMP on the HSCs’ surface is regulated by LRs incorporation [65]. Cytokines such as G-CSF activate PI3K in LRs, leading to the activation of the PI3K/Akt pathway, MT1-MMP embodiment in LRs, and pro-MMP-2 activation. Active MMP-2 modulates the matrix, inactivating SDF-1 and CXCR4 [66] and enhancing pericellular degradation of ECM components such as fibronectin, gelatin, vitronectin, fibrillar collagens, and laminin [67]. These changes result in the release of HSCs of the bone marrow niches across subendothelial membranes and ECM [66] (Figure 2). Accordingly, the incorporation of MT1-MMP in LRs is critical to the HSCs’ mobilization. On the other hand, the disruption of LRs by MβCD or statin inhibits the inclusion of MT1-MMP into LRs, resulting in immobilized HSCs [66].

## 3. HSC Differentiation

**Figure 3:**
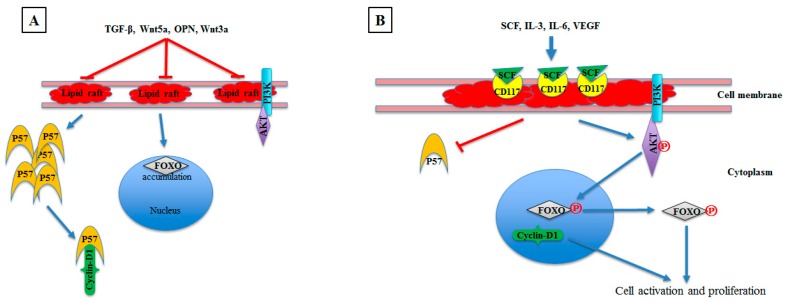
Role of LRs in HSC differentiation. **A**, HSCs rest in hibernation mode in bone marrow niche under the effect of Wnt5a, OPN, Wnt3a, or TGF-β cytokines. These cytokines decrease LRs clustering, show inactive AKT, abundant p57^Kip2^, and nuclear localization of FOXO transcription factors. Abundant p57^Kip2^ binds Cyclin-D1, resulting in inhibition of cell proliferation. **B**, Activation of LR clustering by SCF or IL-3 or IL-6 or VEGF leads to incorporation of CD 117 in the LR cluster, resulting in simulation of the PI3K–Akt–FOXO pathway. Active AKT ℗ phosphorylates FOXO, resulting in translocation of FOXO from the nucleus to the cytoplasm and initiation of HSC cell cycling. LR clustering and CD117 incorporation also reduce P57 abundance, thus increasing Cyclin-D1 translocation into the nucleus with additional initiation of HSC proliferation.

The process of HSC differentiation is an important issue, and revealing its mechanism might open up new avenues for therapeutic strategies. Hematopoietic stem cells rest in hibernation mode in bone marrow niches [68,69]. Occasionally, the HSCs enter the cell cycle after cytokines activation. The fate of these stem cells is determined by secreted molecules or by signals through the cell surface. HSCs in bone marrow niches lack LR clustering and show inactive serine/threonine kinase AKT, abundant p57^Kip2^ cyclin-dependent kinase inhibitor, and nuclear localization of FOXO transcription factors (FOXO1, FOXO3, FOXO4, and FOXO6) [70]. Activation of LR clustering by SCF incorporates receptor tyrosine kinase c-Kit (CD 117) in LR clusters and stimulates the PI3K–Akt–FOXO pathway, which induces HSC activation and cell cycling [71]. On the other hand, the inhibition of LR clustering and induction of p57^Kip2^ expression by TGF-β induces HSC hibernation. In addition, inhibition of LR clustering by methyl–β–cyclodextrin (MβCD) shows inhibition of AKT downstream activity and nuclear localization of FOXO and thus inhibition of HSC proliferation. These findings highlight the importance of LRs in HSC fate [70,72,73] (Figure 3).

The LR status of clustering or diffusion could therefore be an indicator of HSC activation or inhibition, respectively. Other proteins have also been shown to contribute to LR clustering or diffusion and thus to HSC activation or hibernation. For example, Wnt5a, OPN, Wnt3a, and TGF-β decrease LR clustering and cell cycling, whereas IL-3, IL-6, and VEGF activate LR clustering, resulting in CD 117 colocalization with LRs, indicating HSC activation through the PI3K-Akt pathway [71,74,75] (Figure 3).

Prominin-1 (CD133), a membrane glycoprotein associated with LR-membrane vesicles [76], has been identified as a marker of neuronal cells [77], immature hematopoietic stem cells [78,79], and cancer stem cells [80]. CD133 is located in the finger-like projections of the cell membrane of HSCs, where it can bind to cholesterol-containing LRs and is involved in various signaling functions [81,82]. It is also associated with released membrane vesicles in body fluids [76]. CD133 is an important physiological regulator of stem cell expansion and maintenance and has been linked to stem cell fate decisions [83,84]. During the process of differentiation, hematopoietic stem and progenitor cells (HSPCs) release CD133-containing membrane vesicles (CD133-CMV), which are then internalized by feeder cells. These data showed that the CD133-containing LRs may host critical roles in maintaining stem cell properties and their loss or reduction may cause cellular differentiation [85].

The liver X receptors (LXRs) are cholesterol-sensing nuclear receptors which are activated by oxidized derivatives of cholesterol (oxysterols). LXRs induce expression of cholesterol efflux transporter APOE and Abcg1, thus inhibiting cholesterol absorption [86,87,88]. Cholesterol efflux takes place at the cell membrane through LRs [89]. Therefore, expression of APOE and Abca1/g1 in HSCs interrupts membrane LRs, resulting in inhibition of HSC proliferation and differentiation to myeloid cells. On the other hand, APOE and Abca1/g1 knockout mice show upregulation of membrane cholesterol and LR formation that results in HSC monocytosis [90,91]. Thus, LXR modulates LR integrity, which affects the fate of HSCs.

LRs have been described in many different cell types, including HSCs, to be the platforms for signaling molecules implicated in the management of cell differentiation. LRs have been investigated for their involvement in the blockage of neutrophil differentiation during *Clostridium perfringens* infection. The LR marker GM1 ganglioside was found to be reduced with neutrophil differentiation and increased with α-toxin (from *Clostridium perfringens* type A) treatment of bone marrow cells. Also, infection of *Clostridium perfringens* type A increased the GM1 expression at cell surface of myeloid cells. These data were confirmed by disruption of LRs by MβCD that resulted in the blockage of neutrophil differentiation [92], indicating direct involvement of LR content and integrity in neutrophil fate.

The effect of vesicles on the fate of HSCs is commonly discussed in many research papers, indicating the major role of these vesicles in HSC differentiation. The entry of extracellular vesicles is mediated through LRs. For example, megakaryocytic microparticles, small membrane vesicles derived by budding from the cell membrane of megakaryocytes, can fuse into the cell membrane or get endocytosed into hematopoietic and progenitor stem cells through micropinocytosis and LRs. This process results in the differentiation of HSPCs into megakaryocytes, indicating the coordinated role of LRs and extracellular vesicles on HSC differentiation [93].

## 4. Summary

LRs are membrane platforms that regulate cell signaling and differentiation through protein–protein and protein–lipid interactions in hematopoietic stem cells. LR clustering or interruption is the main effector on HSCs differentiation, mobilization, and hibernation. The activation of LR clustering by SCF, IL-3, IL-6, and VEGF initiates HSC activation, while the inhibition of LR clustering by Wnt5a, OPN, Wnt3a, and TGF-β results in HSC hibernation. LXRs interrupt LR integrity, resulting in inhibition of HSC differentiation. However, CD133-containing LRs may be responsible for the maintenance of HSC properties and their loss may result in differentiation. On the other hand, endocytosis of extracellular vesicles through LRs enhances HSC-specific differentiation. For example, the internalization of megakaryocytic microparticles through LRs into HSPCs results in the differentiation of HSPCs into megakaryocytes. LRs are also involved in HSC mobilization. For example, disruption of LRs by PLC-β2 in ECM results in HSC mobilization. In addition, incorporation of MT1-MMP into LRs, which enhances the degradation of the connection between HSCs and ECM, results in the release of HSCs.
